# SOCS, Intrinsic Virulence Factors, and Treatment of COVID-19

**DOI:** 10.3389/fimmu.2020.582102

**Published:** 2020-10-23

**Authors:** Howard M. Johnson, Alfred S. Lewin, Chulbul M. Ahmed

**Affiliations:** ^1^ Department of Microbiology and Cell Science, University of Florida, Gainesville, FL, United States; ^2^ Department of Molecular Genetics and Microbiology, University of Florida, Gainesville, FL, United States

**Keywords:** antiviral peptide, cytokine signaling, tyrosine kinase, COVID-19, SARS-CoV-2

## Abstract

The suppressor of cytokine signaling (SOCS) family of intracellular checkpoint inhibitors has received little recognition compared to other checkpoint inhibitors. Two members of this family, SOCS1 and SOCS3, are indispensable, since SOCS1 knockout in mice results in neonatal death due to interferon gamma (IFNγ) induced inflammatory disease, and SOCS3 knockout leads to embryonic lethality. We have shown that SOCS1 and SOCS3 (SOCS1/3) function as virus induced intrinsic virulence factors for influenza A virus, EMC virus, herpes simplex virus 1 (HSV-1), and vaccinia virus infections. Other viruses such as pathogenic pig enteric coronavirus and coronavirus induced severe acute respiratory syndrome (SARS) spike protein also induce SOCS virus intrinsic virulence factors. SOCS1/3 exert their viral virulence effect *via* inhibition of type I and type II interferon (IFN) function. Specifically, the SOCS bind to the activation loop of receptor-associated tyrosine kinases JAK2 and TYK2 through the SOCS kinase inhibitory region (KIR), which inhibits STAT transcription factor activation by the kinases. Activated STATs are required for IFN function. We have developed a small peptide antagonist of SOCS1/3 that blocks SOCS1/3 inhibitory activity and prevents virus pathogenesis. The antagonist, pJAK2(1001-1013), is comprised of the JAK2 activation loop, phosphorylated at tyrosine 1007 with a palmitate for cell penetration. The remarkable thing about SOCS1/3 is that it serves as a broad, simple tool of perhaps most pathogenic viruses to avoid innate host IFN defense. We suggest in this Perspective that SOCS1/3 antagonist is a simple counter measure to SOCS1/3 and should be an effective mechanism as a prophylactic and/or therapeutic against the COVID-19 pandemic that is caused by coronavirus SARS-CoV2.

## Introduction

Global public health is under siege as a result of a coronavirus infectious pandemic disease that may have originated in Wuhan, China in late 2019 (1, 2), thus the acronym (COVID-19). The causative viral agent of COVID-19, SARS-CoV2, is a variant of the 2002/2003 pandemic coronavirus, SARS-CoV, where the acronym SARS represents severe acute respiratory syndrome (1, 2). Another relative of SARS that was responsible for SARS-like syndrome epidemic in the Middle East in 2012 has the acronym MERS-CoV (2). Thus, there are three relatively recent pandemics/epidemics involving members of the betacoronavirus family (2). It therefore seems reasonable that additional betacoronavirus variants or strains will cause some future virus induced pandemic.

Coronaviruses are not newly discovered respiratory pathogens for humans as several strains are commonly involved in “head cold” type of illness (3). The SARS viruses, however, are a special case, particularly in the context of seasonal influenza virus respiratory disease (4, 5). It is anticipated that at some point these two groups of viruses will cause serious health problems at the same time. Thus, in this Perspective, we will address these health problems in the context of a recently discovered virus induced intrinsic virulence system that plays a key role in virus pathogenesis. The implication of this discovery is that a common or single antiviral could be an effective preventative/therapeutic against both SARS-CoV2 and influenza viruses.

The virus induced non-specific intrinsic virulence system consists of checkpoint inhibitors called suppressors of cytokine signaling (SOCS) (6–8). SOCS consist of eight intracellular proteins, SOCS1 to SOCS7, and cytokine-inducible Src homology 2 protein, CIS. It is SOCS1 and 3 (SOCS1/3) that function as virulence factors, but that is not their evolutionary purpose, as both are required for viability. Knockout of SOCS1 in mice results in neonatal death, primarily due to unregulated inflammation caused by gamma interferon (IFNγ) (9). SOCS3 knockout results in embryonic death (10). It is the neonatal inflammation induced death that is intriguing. This is stark evidence that these mice are not protected by other checkpoint inhibitors absent SOCS1. Thus, SOCS1 is a key but under-recognized immune checkpoint inhibitor.

**Figure 1:**
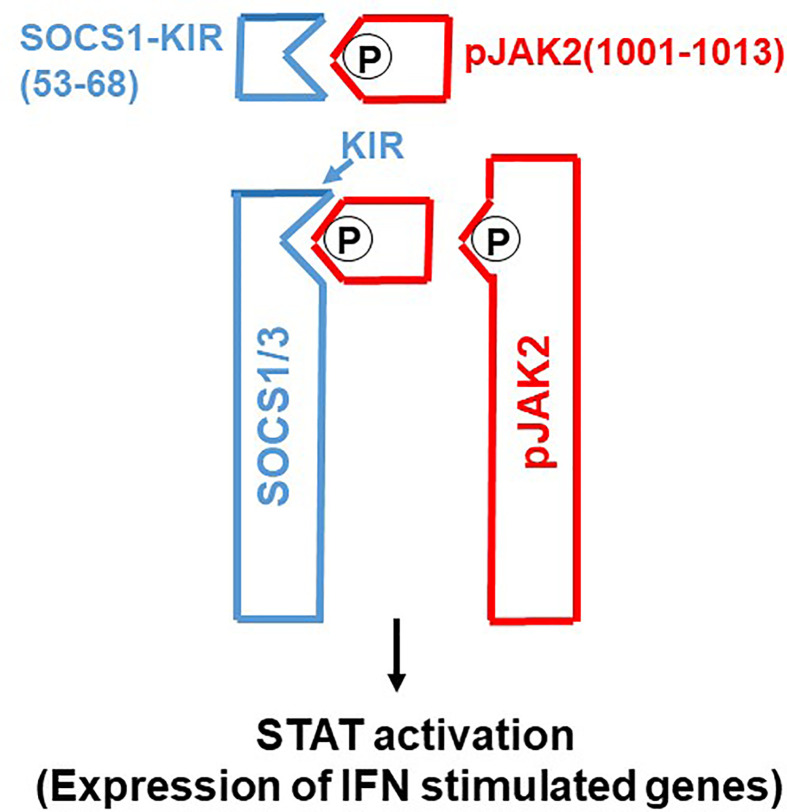
SOCS1/3 antagonist. Scheme of how SOCS1/3 antagonist, pJAK2(1001-1013), inhibits SOCS1 and 3 blockage of activation of JAK2 (or (TYK2) for induction of antiviral activity of interferon. P, indicates phosphorylation.

Molecular tools such as gene transfection and siRNA have played a major role in our functional understanding of SOCS proteins where a key functional domain of 12 amino acids called the kinase inhibitory region (KIR) has been identified on SOCS1 and SOCS3 (6). KIR plays a key role in inhibition of the JAK2 tyrosine kinase, which in turn plays a key role in cytokine signaling. A peptide corresponding to KIR (SOCS1-KIR) bound to the activation loop of JAK2 and inhibited tyrosine phosphorylation of STAT1α transcription factor by the kinase. Cell-internalized SOCS1-KIR is a potent therapeutic in experimental allergic encephalomyelitis (EAE), a mouse model of multiple sclerosis and showed promise in a psoriasis model and a model of diabetes-associated cardiovascular disease (11–13). By contrast, a peptide, pJAK2(1001-1013), that corresponds to the activation loop of JAK2 is a SOCS1 and SOCS3 inhibitor *via* KIR binding (7). In sections below, we show the power of SOCS1/3 antagonist as an effective therapeutic against the SOCS1 and SOCS3 virus induced virulence factors (**Figure 1**).

## SOCS and Other Members of the Checkpoint Inhibitor Family

Checkpoint inhibitors are a complex network of cells, soluble factors, and cell-associated proteins that govern and control the immune response. As indicated with SOCS1, they prevent the immune system from over-responding to both foreign and self-antigenic stimuli (6, 7). Specifically, the checkpoint inhibitor families consist of, but is not limited to, members of SOCS family, program death 1 cell protein (PD-1) and its cellular ligand, PD-1L, and cytokine T lymphocyte antigen 4 (CTLA-4) (14–17). CTLA-4 is a key effector molecule involved in Treg function (15, 16). There is cross-talk between SOCS1 and Forkhead box (FoxP3) positive natural or constitutive Tregs which is important in peripheral Treg homeostasis (18). Manipulation of PD-1 and CTLA-4 with inhibitory monoclonal antibodies to reduce their immune inhibition is widely used in immunotherapy of various cancers with some success (19, 20). Thus, there is a complex network of coordinated interactions among these various immune checkpoint inhibitors that is important in immune homeostasis that is ripe for manipulation in immunotherapeutic approaches to cancer and immune deficiency disorders. Manipulation of SOCS1 and SOCS3 should play a key role in viral infections, particularly those caused by viruses that are associated with respiratory diseases.

## SOCS1/3 and Various Viral Infections

There is considerable evidence that SOCS1 and SOCS3 play important roles in viral immune evasion involving a broad range of viruses. In fact, it is our contention that SOCS1/3 is broadly hijacked by viruses to function effectively as viral virulence factor(s). Coronavirus transmissible gastroenteritis virus (TGEV), for example, uses SOCS1 and SOCS3 to evade type I interferons (IFN-I), but that does not actually affect IFN induction (21). Replication of coronaviruses is closely tied to the endoplasmic reticulum (ER); ER stress occurs as a result of TGEV and other coronavirus infection of cells. The coronavirus TGEV study defined the complex events involved in blocking IFN-I in host defense (21). Details of the mechanism of TGEV induction of SOCS1 and SOCS3 are presented below. Although TGEV is an alphacoronavirus, while SARS-CoV2 is in the betacoronavirus family, both viruses can attack the gut, which is significant in the approach of attacking SARS-CoV2 in terms of SOCS1/3 virulence factors (21).

An immortalized chicken cell line provides a particularly interesting example of the relationship between constitutively elevated SOCS1 and innate immune responses (22). In a comparison of immortalized chicken DF-1 cell line and primary chicken embryo fibroblasts (CEFs), DF-1 cells had 16-fold higher levels of SOCS1 than did CEFs. Consistent with the SOCS pattern, treatment of these cells with chicken IFNα resulted in decreased expression of IFN-stimulated genes in DF-1 cells, compared to CEFs. Similarly, an attenuated chicken bursal disease virus, PBF98, had a significantly higher yield in IFN treated DF-1 cells than in CEFs. The authors found that SOCS1 mediated these effects, but that the SOCS box domain was not required for the SOCS1 effect. Specifically, siRNA inhibition of SOCS1 mRNA with wild type or SOCS1 box deletion siRNA constructs similarly inhibited the SOCS1 effects in DF-1 cells. As indicated, we have developed a peptide that corresponds to the KIR region of SOCS1, which we designated SOCS1-KIR (18, 23). The mimetic is the complement or mirror image of SOCS1/3 antagonist, indicating that KIR is the binding site on SOCS1 and SOCS3 for the antagonist (23). As indicated, SOCS1-KIR has been used to successfully treat autoimmune disease in mouse models (11–13). The studies with the DF-1 cells suggest that the KIR region of SOCS1 was sufficient to mediate the SOCS1 effects in these cells.

**Figure 2:**
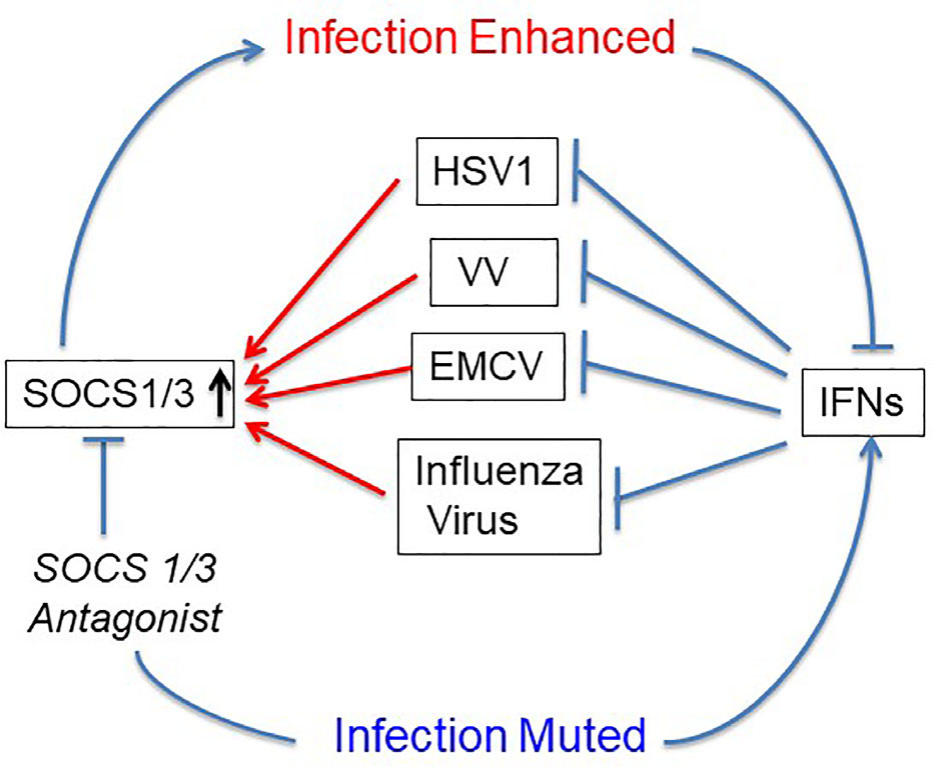
SOCS1/3 blocks IFN activity. Viruses with different mechanisms of replication have been shown to upregulate or use constitutive SOCS1/3 as virulence factors. SOCS1/3 antagonist peptide blocks the SOCS, thus freeing the interferons (IFNs) to inhibit virus replication. HSV-1, herpes simplex virus 1; VV, vaccinia virus; EMCV, encephalomyocarditis virus.

The first use of SOCS1/3 antagonist in a virus infection involved the double-stranded DNA virus, herpes simplex virus (HSV-1) (24). Keratinocytes were refractory to IFNγ induction of an antiviral state to HSV-1 infection, while IFNγ did induce an antiviral state in fibroblasts (L929). RT-PCR showed that HSV-1 induced a 4-fold increase in SOCS1 mRNA in keratinocytes, but only a negligible increase in fibroblasts. A similar pattern was observed at the level of SOCS1 protein. Treatment of the keratinocytes with palmitated (cell penetrating) pJAK2(1001-1013) rendered both an antiviral state as well as a synergistic effect when combined with IFNγ treatment. These findings with HSV-1 were rather remarkable. The question is whether the HSV-1 findings with antagonist applies to other viruses. SOCS showed similar antagonism against vaccinia virus and encephalomyocarditis virus (EMCV), both in culture and in mouse models of lethal virus infection (25). Vaccinia virus (VV), like HSV-1 is a double stranded DNA virus, but with its own set of genetic complexities. VV replicates in the cytoplasm, while HSV-1 replicates in the nucleus. EMCV is a picornavirus whose genome is plus-stranded RNA like that of coronaviruses (25). It is particularly impressive that pJAK2(1001-1013) protected VV and EMCV injected mice against acutely lethal doses of viruses. This provided preclinical evidence of efficacy of the SOCS antagonist as an antiviral (**Figure 2**).

pJAK2(1001-1013) also possessed potent adjuvant activity at both humoral and cellular levels against a protein antigen, bovine serum albumin (BSA) (25). Potential significance of this observation is that soluble BSA is not particularly effective at inducing a cell mediated response. In conjunction with the adjuvant properties, the SOCS antagonist also enhanced poly(I:C) activation of TLR3.

In addition to the above viruses, the SOCS1/3 antagonist also inhibited a type A influenza virus (26), a negative strand segmented RNA virus (27). This virus is particularly important in the context of the current pandemic with SARS-CoV2. SOCS1 has been shown to be an influenza virus-induced virulence factor that enhances infection of cells. The antagonist was protective in cell culture and in influenza virus PR8 lethally infected C57BL/6 mice.

The SOCS antagonist also prevented adverse morbidity as assessed by parameters, such as weight loss and drop in body temperature, and showed potent induction of both the cellular and humoral immune responses to the influenza virus candidate universal antigen matrix protein 2 (M2e) (26). The SOCS antagonist, thus protected mice against lethal influenza virus infection and possessed potent adjuvancy against the M2e candidate influenza virus universal vaccine antigen. Thus, an inhibitor of both coronavirus and influenza A virus could reduce the complexity of respiratory infections by these two viruses. Dealing with these two viruses at the same time is a challenge that we most likely will face.

There are several other virus strains or types that, like the viruses above, use SOCS1 or SOCS3 as a virulence factor. These include at least dengue virus (plus strand RNA (28), Zika virus (plus strand RNA) (29), West Nile virus (also plus strand RNA) (30), and Ebola virus (31). The role that SOCS1/3 plays in the pathogenesis of all these viruses as well as the future challenges of potential epidemics/pandemics that they and other viruses may cause places particular importance on attacking the SOCS virulence factors.

## SOCS1/3 Antagonist as a Therapeutic for COVID-19

As indicated, TGEV enteric coronavirus infection in pigs induced SOCS1 and 3 (SOCS1/3) intrinsic virulence factors, which played a role in virus immune evasion (21). In addition, the virus attacks a microRNA, miR-30-5p, that regulates the expression of SOCS1 and SOCS3 at the level of mRNA and protein induction. It was shown that the evasion occurs as a result of virus activation of an endoplasmic reticulum RNase that degrades miR-30-5p, resulting in the release of SOCS1/3 mRNA to produce SOCS1/3 proteins, which in turn block JAK2 and TYK2 tyrosine activation. Blocked TYK2 results in failure of activation of the STAT transcription factors that mediate type I IFN activity (32).

Rather than inhibition of induction of SOCS1/3, the SOCS1/3 antagonist, as indicated, blocks SOCS function by binding to the KIR region of SOCS1 and SOCS3. Thus, JAK2 and TYK2 are activated for subsequent activation of the STAT transcription factors that mediate IFNγ and type I IFN antiviral function, respectively.

As referenced, SARS-CoV and SARS-CoV2 are both betacoronaviruses, so while the latter has not been examined for SOCS1/3 induction, there is evidence for such induction by SARS virus, SARS-CoV. Transfection of a human B cell lymphoma (Toledo) with recombinant baculovirus vAtEpG5688 expressing amino acids 17-688 of SARS-CoV spike protein on the surface of the envelope induced over a 4-fold increase in SOCS3 over 48 h (33). In another study, infection of heterogeneous human epithelial colorectal cancer cells (Caco2) with SARS-CoV also induced SOCS3 but to a lesser extent than the B cell lymphoma cell line (34). None of these limited studies focused in particular on the SOCS proteins, and the findings were not interpreted in the context of the role of SOCS3 in virus infection, as was done in the case of TGEV.

No such SOCS studies have been done with SARS-CoV2, not to mention in the context of SOCS1/3 virus-induced intrinsic virulence factors. Given the current circumstances of empirical rather than mechanistic approaches to treating COVID-19, focus on SOCS1/3 would seem both reasonable and rational.

For reasons not fully understood, a subset of the population seems particularly susceptible to a life threatening severe form of COVID-19 (5, 35). In the US, African-Americans are more likely to die of COVID-19 than are Americans of European descent (36, 37). Obesity coupled with type 2 diabetes, has also been shown to be a risk factor for severe COVID-19 in terms of morbidity and mortality (38, 39). Specific indicators beyond the general observation that overweight people are less healthy than non-fat people have not been definitively identified to suggest enhanced susceptibility to COVID-19 in these groups, but one meta-analysis indicates increased expression of ACE2, the receptor for SARS-CoV2 in subcutaneous and visceral adipose tissue (40). If one looks at COVID-19 in the obese, there may be a SOCS connection. Specifically, SOCS3 has clearly been shown to be associated with type 2 diabetes, and in fact is positively correlated with insulin resistance (41). Furthermore, the incidence of influenza is higher in obese compared to non-obese individuals (42). Thus, a virus virulence factor, SOCS3, is expressed at a higher level in the obese, who are at greater risk of the flu than are non-obese individuals. It would be of considerable interest to therefore determine the role of elevated SOCS3 in the obese in their greater susceptibility to COVID-19, influenza, and other viral diseases in insulin resistant individuals.

## SOCS1/3 Antagonist and Severe COVID-19

The discussion here addresses the potential downside of SOCS1/3 antagonist as virus inhibitor, with potential exacerbation of the inflammatory condition that drives severe COVID-19. COVID-19 begins with SARS-CoV2 replication in the upper respiratory tract. Currently (October 1, 2020), the USA has over 7,184,000 known infections with more than 208,000 deaths. The fatality rate is thus approximately 3%, which is 30 times greater than that of seasonal influenza (43). Infection can be asymptomatic or of varying degrees of severity. Severe COVID-19 that can result in death is characterized by pulmonary as well as multi-organ disease involving the heart, vascular system, kidneys as well as other organs and tissues (44–48). The cell receptor for SARS-CoV2 is angiotensin-converting enzyme 2 (ACE2), which is widely expressed on endothelial and smooth muscle cells in almost all organs (49). The lungs of patients who have died of COVID-19 show diffuse alveolar damage, severe endothelial injury associated with intracellular virus, and disrupted cell membranes (44). Pulmonary vessels show widespread thrombosis and microangiopathy. The inflammatory condition and thrombosis extends in varying degrees to the heart, gut, and kidneys. Importantly, SARS-CoV2 presence is associated with pathologic profile. The inflammatory illness in children is consistent with the vascular cell target of SARS-CoV2 (50). SARS2-CoV2 infection in children results in autoimmune and autoinflammatory responses that are termed as pediatric inflammatory multisystem syndrome (PIMS) or multisystem inflammatory syndrome in children (MISC-C) (45, 51, 52). The immune and inflammatory cells as well as inflammatory cytokine profile has resulted in the phrase “cytokine storm” in the severe disease. Particular interest is placed on the cytokines of interleukin (IL)-6, IL-1, and tumor necrosis factor (TNF)-α (53–57). A reasonable question pertaining to the SOCS1/3 antagonist is whether it would exacerbate the cytokine storm and other hyperimmune aspects of COVID-19, particularly so as there are considerable efforts to improve disease outcomes by anti-inflammatory and anti-clotting approaches (46, 47, 49, 54, 55). We will address this after briefly presenting some current approaches to COVID-19.

Antiviral drug, remdesivir, was introduced early and has shown slight improvement in severe but non-fatal COVID-19 (58). Type I IFNs, IFNα, and IFNβ, have shown antiviral and clinical effects against SARS-CoV2, and COVID-19, but it is early in their clinical use to draw firm conclusions (59–62). An unusual aspect of SARS-CoV2 infection is the limited induction of type I IFN following infection. This suppression may be mediated in part by the viral ORF3b protein that suppresses the induction of interferon (63). Paradoxically, inhibition of a receptor-associated tyrosine kinase called JAK1 that is required for IFN signaling, has also been shown to have some clinical efficacy against COVID-19 (62, 64, 65). The paradox may be related to mechanism of action. IFN inhibits virus replication, while JAK1 inhibitors probably inhibit some aspects of the cytokine storm. As with IFN, the JAK family is also involved in mechanisms of cytokine signaling in general and thus the JAK inhibitors may block cytokine storm cytokines. The use of monoclonal antibodies to inhibit the relevant cytokines in severe COVID-19, however, have not been particularly effective (66). In this regard, another antibody approach, the use of convalescent serum with neutralizing antibodies to SARS-CoV2 has also not significantly improved severe COVID-19 in patients in spite of high profile political pronouncements and promotion (67). There is limited indirect and direct evidence that T regulatory cells (Tregs) have some therapeutic efficacy in severe COVID-19. Il-2 induced Tregs as well as cord blood derived Tregs appeared to rescue patients that were on ventilators in limited studies (53, 68). There are other prospective treatments in the pipeline, but the patterns are similar to those of the above approaches. In fact, meta-analyses sequential analyses found that beyond modest certainty of evidence supporting dexamethasone and remdesivir, no other evidence based treatment for COVID-19 currently exists (67).

As indicated, it is reasonable to have concerns that SOCS1/3 antagonist could exacerbate the inflammatory reaction due to adaptive immune cells, innate immune cells, and the interleukins associated with the cytokine storm. SOCS1 and SOCS3 are key checkpoint inhibitors or regulators of the immune system and they are the targets of SOCS1/3 antagonist. It is worthwhile, however, to remember that SARS-CoV2 is present in the cells of the lesions associated with severe COVID-19. Thus, it is paramount to attack the virus before it overwhelms the lungs and other tissues and initiates the cytokine storm. It is our thesis that SOCS1/3 antagonist can serve the function of limiting SARS-CoV2 load and that such limiting should reduce the incidence of the self-destructive host defense that is reflected by the cytokine storm.

## Author Contributions

CA designed and performed experiments. HJ and AL supervised and wrote the manuscript. All authors contributed to the article and approved the submitted version.

## Funding

This work was partly supported by the NIH grant R01 AI056152 (HJ), Shaler Richardson Professorship Endowment (AL).

## Conflict of Interest

The authors declare that the research was conducted in the absence of any commercial or financial relationships that could be construed as a potential conflict of interest.
